# Protein misfolding and mitochondrial dysfunction in glaucoma

**DOI:** 10.3389/fcell.2025.1595121

**Published:** 2025-04-25

**Authors:** Arunkumar Venkatesan, Audrey M. Bernstein

**Affiliations:** ^1^ Department of Ophthalmology and Visual Sciences, SUNY Upstate Medical University, Syracuse, NY, United States; ^2^ New York VA Healthcare, Syracuse VA Medical Center, Syracuse, NY, United States

**Keywords:** er stress, upr, autophagy, mitochondrial dysfunction, POAG, NTG, XFG, glaucoma

## Abstract

Glaucoma is a leading cause of irreversible blindness worldwide. Elevated intraocular pressure caused by restricted outflow of the aqueous humor leads to the degeneration of retinal ganglion cells (RGCs) and their axons. Emerging evidence suggests that pathological mechanisms relating to protein folding and mitochondrial dysfunction are significant factors in the disease onset of different types of open-angle glaucoma. In this review, we discuss these defects in three distinct types of open-angle glaucoma: primary open-angle glaucoma (POAG), normal tension glaucoma (NTG), and exfoliation glaucoma (XFG). Genetic mutations linked to the previously mentioned open-angle glaucoma, including those in myocilin (MYOC), optineurin (OPTN), and lysyl oxidase 1 (LOXL1), disrupt protein folding and homeostasis, leading to endoplasmic reticulum stress, activation of the unfolded protein response and impaired autophagic protein degradation. These factors contribute to trabecular meshwork and retinal ganglion cell apoptosis. In addition to protein folding defects, mitochondrial dysfunction is also associated with the progression of trabecular meshwork damage and the death of RGCs. Factors such as oxidative stress, an altered mitochondrial fission-fusion balance, and mitophagy dysregulation make RGCs vulnerable and contribute to optic nerve degeneration. The crosstalk between protein folding and mitochondrial defects in glaucoma underscores the complexity of disease pathogenesis and offers potential targets for therapeutic intervention. Strategies aimed at restoring protein homeostasis, enhancing mitochondrial function, and mitigating cellular stress responses hold promise for neuroprotection in glaucoma.

## Introduction

Glaucoma is a heterogeneous group of disorders characterized by gradual loss of peripheral-to-central vision due to optic neuropathy and the death of retinal ganglion cells (RGCs), leading to irreversible vision loss (Quigley, 1999; Allingham et al., 2009; Kwon et al., 2009). It is a leading cause of irreversible blindness, with over 60 million people affected worldwide, and its prevalence is estimated to increase to 111.8 million by 2040 (Tham et al., 2014). Elevated intraocular pressure (IOP) (≥22 mmHg) is a major risk factor for RGC loss and is often, but not always, associated with glaucoma. The trabecular meshwork (TM) and Schlemm’s canal (SC) in the aqueous humor (AH) outflow pathway is a tissue that regulates IOP via the effective discharge of AH produced by the ciliary body. However, an imbalance between the production and outflow of AH causes elevated IOP within the anterior chamber of the globe, and adverse mechanical pressure on the optic nerves (ON) causes optic neuropathy. This loss of RGCs is associated with structural changes to the optic nerve head (ONH) and loss of visual field (Kwon et al., 2009; Alvarado et al., 1984).

Glaucoma can be divided into primary and secondary forms. Primary open-angle glaucoma (POAG) is the most common type, in which the anterior chamber angle appears normal with increased IOP and no other underlying disease. Juvenile open-angle glaucoma (JOAG) presents at a much earlier age and is also characterized by higher IOP and a more severe progression compared to adult-onset POAG. Normal tension glaucoma (NTG) is a type of POAG marked by ON degeneration despite normal IOP (Rezaie et al., 2002). Exfoliation glaucoma (XFG) is a severe form of secondary glaucoma, with the elevation of IOP associated with the deposition of white, flaky aggregates in the anterior chamber of the eye (Dietze et al., 2024). Glaucoma is a multifactorial disease involving multiple genes and environmental factors in the disease pathogenesis (Wiggs, 2007). Recent advancements in genome-wide association studies (GWAS) and next-generation whole exome sequencing have broadened our understanding of the risk variants linked to various types of glaucoma (Huang et al., 2018). However, these genetic risk variants are often flipped in different populations, complicating the understanding of the disease (Wiggs and Pasquale, 2017).

Proteostasis is essential for cellular survival, and any perturbations in protein homeostasis can lead to diseases such as neurodegenerative, metabolic, and cardiovascular disorders (Balch et al., 2008). The endoplasmic reticulum (ER) organelle is a protein quality control system of the secretory pathway proteins, permitting only correctly folded proteins to exit the organelle. In contrast, misfolded proteins are degraded through ER-associated degradation (ERAD) and autophagy (Poothong et al., 2021). Mitochondria is a dynamic organelle and critical for cellular energy metabolism and apoptosis. Mitochondrial dysfunction is linked to a wide range of neurodegenerative pathologies, including Alzheimer’s disease, Parkinson’s disease, and amyotrophic lateral sclerosis (ALS) (Lin and Beal, 2006; Johri and Beal, 2012; Chen et al., 2023; Klemmensen et al., 2024). The close connection between ER and mitochondria is crucial for maintaining cellular homeostasis, particularly under stress conditions, as miscommunication could lead to metabolic diseases (Senft and Ronai, 2015). This review focuses on the protein folding and mitochondrial dysfunction defects caused by the pathogenic variants of POAG, NTG, and XFG glaucoma and provides new insights for targeted therapeutics.

### Protein folding defects and neurodegeneration in glaucoma

**FIGURE 1:**
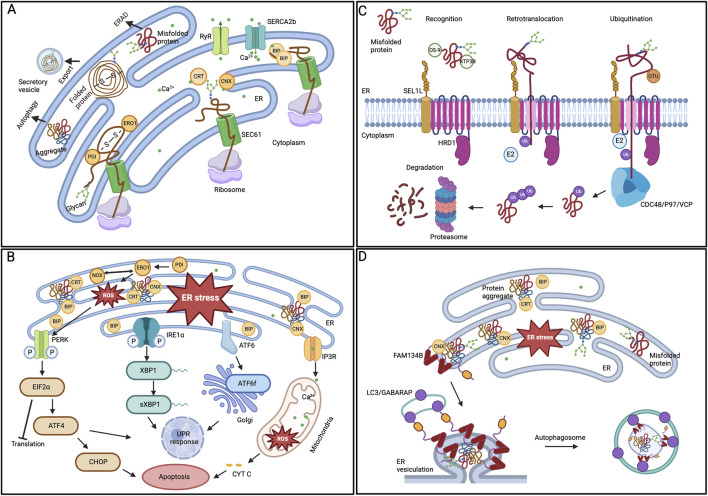
ER protein quality control mechanisms **(A)** Protein folding in ER lumen. Newly synthesized proteins that are translocated to the ER by the SEC61 translocon are folded by various ER chaperones. While the CRT/CNX cycle aids glycoproteins, PDI oxidizes cysteine residues to promote disulfide bond formation. Properly folded proteins can exit the organelle in vesicles. **(B)** ER stress and UPR activation. BIP removes its interaction from the three UPR signaling pathways in response to ER stress, initiating adaptive signaling. The PERK, IRE1α, and ATF6 pathways parallelly activate downstream modulators and regulate the gene expression to restore ER homeostasis. However, prolonged ER stress leads to the activation of cellular apoptosis. **(C)** ERAD degradation of substrate proteins. The ERAD substrates recognized by the chaperones are ubiquitinated and retro-translocated to the cytoplasm by the SEL1-HRD1 complex. In the cytoplasm, the CDC48/P97/VCP ATPase trims and re-ubiquitinates the substrates before being handed over to the proteasome for degradation. **(D)** ER-phagy mechanism. One of the ER-phagy receptors, FAM134B, identifies the ER subdomain that contains protein aggregates, aided by the chaperone CNX, for selective degradation. The receptor’s oligomerization at the ER sheets’ edge induces curvature in the ER membrane. Further, the interaction between the ER-phagy receptor and LC3/GABARAP facilitates the sequestration of ER regions within autophagosomes.

The ER is a dynamic signaling organelle where proteins are co-translationally folded by the ER protein quality control (ERQC) mechanism with the assistance of resident glycan (calnexin (CNX), calreticulin (CRT)) and HSP (GRP78/BIP, GRP94) family chaperones. These proteins are stabilized through post-translational modifications (PTMs) such as N-glycosylation, lipidation, and disulfide bond formation (guided by PDI and ERO1), and only those that achieve their native conformation are exported via the secretory pathway. The ER also acts as an intracellular storage site for Ca^2+^ (ERCa^2+^), crucial in influencing protein folding efficiency (Figure 1A) (Venkatesan et al., 2021). Proteins that do not achieve functional native conformation are prone to aggregation, where the misfolded monomer self-associates to form oligomers, further extending to create amyloid fibrils with a beta-sheet structure and well-organized amyloid plaques (Poothong et al., 2021). The accumulation of misfolded protein aggregates and unfolded proteins disrupts ER homeostasis, leading to stress in the ER and the development of diseases known as proteinopathy (Ganguly et al., 2017). As an adaptive reaction, ER stress triggers a sequence of cellular stress responses, including the unfolded protein response (UPR), pathways related to oxidative stress, and inflammatory responses to restore proteostasis and cellular homeostasis. BIP regulates UPR and activates three key signaling cascades: PERK, IRE1α, and ATF6 (Figure 1B). These cascades initiate a coordinated response, leading to distinct action branches that restore ER homeostasis by reducing protein synthesis, enhancing chaperone protein expression, and degrading misfolded proteins through ERAD. To alleviate ER stress, the PERK-ATF4-CHOP signaling cascade halts protein translation by activating the eIF2α and triggers apoptosis if the stress endures for an extended period. The IRE1α pathway, via the *XBP1* mRNA splicing, activates the promoters of genes related to ERQC and ERAD to induce ER homeostasis as an adaptive response. The ATF6 also activates an adaptive signaling pathway after being cleaved in the Golgi apparatus (Walter and Ron, 2011). When proteins are irreversibly misfolded and cannot be resolved through UPR pathways, the ubiquitin-proteasome-dependent ERAD pathway clears the abnormal proteins (Figure 1C). The ERAD pathway comprises four steps: recognizing misfolded proteins through the SEL1L-HRD1 translocation complex, retrotranslocating substrates to the cytosol using the cdc48/p97/VCP AAA-ATPase, re-ubiquitinating clients in the cytosol, and degrading them via proteasomes (Christianson and Carvalho, 2022). However, some large protein aggregates resistant to ERAD are degraded through lysosome-mediated autophagy. ER-phagy, a form of selective autophagy, uses specific ER membrane receptors, such as FAM134B, to identify sub-domains of the ER that contain misfolded aggregates, which are engulfed by autophagosomes through their interaction with LC3 (Figure 1D) (Mochida and Nakatogawa, 2022).

While the pathogenesis of glaucoma remains unclear, chronic ER stress induction is associated with TM and RGC dysfunction and results in increased IOP in both animal POAG glaucoma models and patients. Healthy TM cells are vulnerable to oxidative stress; for instance, exposure to tetra-butyl hydroperoxide triggers ATF4-mediated ER stress. Further activation of the UPR mediators eIF2α and CHOP triggers apoptosis and inflammatory cytokine production and contributes to IOP elevation (Ying et al., 2021). Additionally, inducing ER stress by overexpressing ATF4 led to TM dysfunction, resulting in elevated IOP and glaucomatous neurodegeneration in a mouse glaucoma model (Kasetti et al., 2020). Interestingly, markers of ER stress are found to be upregulated in TM tissues from patients with POAG. This heightened ER stress triggers the activation of oxidative stress and the PERK arm of the UPR pathway, leading to increased levels of ATF4 and CHOP (Peters et al., 2015). This ER stress and activation of UPR could be one of the causes of dysregulated autophagy in glaucomatous TM cells (Porter et al., 2015). Likewise, the involvement of neuronal ER stress and CHOP activation in RGC death has been reported in chronic glaucoma models (Doh et al., 2010; Hu et al., 2012).

Prolonged activation of the UPR often triggers autophagy to restore homeostasis. Although the involvement of autophagy in ocular hypertension (OHT) and glaucoma is under investigation, alterations in autophagy vary by tissue type. While the anterior chamber tissues of the eye exhibited lower autophagic flux, the posterior chamber ON tissues demonstrated higher levels of autophagy in a DBA/2J (D2) spontaneous glaucoma mouse model (Hirt et al., 2018). Interestingly, autophagy deficiency with the gene knockout of *Atg4b*, a crucial autophagy gene involved in LC3 processing and autophagosome formation in chronic IOP elevation mouse models, resulted in normal IOP and protected against RGC death (Dixon et al., 2023). However, the role of autophagy is complicated as reduced autophagic flux is also associated with certain myocilin (MYOC) mutants that lead to protein aggregation, ER stress, UPR activations, and subsequent TM dysfunction (Kasetti et al., 2021; Yan et al., 2024).

In glaucoma, several lines of evidence indicate that genetic components play a crucial role. Multiple studies are increasingly reporting the involvement of several genetic variants in the pathogenesis of POAG, NTG, and XFG (Wiggs and Pasquale, 2017; Tirendi et al., 2023). Further research on these genetic risk variants has yielded new insights into the underlying molecular mechanisms relevant to glaucoma. In the following section, we will explore the protein folding defects caused by glaucoma risk variants, their impact on TM dysfunction, and the survival of RGCs in various types of open-angle glaucoma.

### Myocilin

Myocilin (MYOC) is commonly expressed in various ocular and non-ocular tissues. The TM was reported to show the highest level of myocilin expression among ocular tissues (Adam et al., 1997). Myocilin expression was also explicitly induced by stress conditions such as mechanical stretch, TGF β , and dexamethasone treatment (Tamm et al., 1999). Myocilin is a secreted glycoprotein encoded by the *MYOC* gene (Nguyen et al., 1998; Caballero and Borras, 2001) composed of an N-terminal coiled-coil (CC) region, which includes the leucine zipper (LZ) motifs. An unstructured linker connects this region to the C-terminal olfactomedin (OLF) homolog domain, which has a high-affinity Ca^2+^ binding site (Kubota et al., 1997; Donegan et al., 2012). The wild-type (WT) MYOC is often observed as a 55/57-kDa doublet in both TM cell lysates and in AH, indicating the protein’s N-glycosylation at amino acid Asn57 (Shepard et al., 2003; Gobeil et al., 2004). However, the importance of MYOC N-glycosylation is not yet known. MYOC is secreted via the ER-Golgi secretory pathway. While the N-terminal CC region is sufficient to target the protein to the Golgi apparatus-mediated secretory compartment, the OLF domains are necessary for efficient secretion to the extracellular space (Stamer et al., 2006). The CC region shares the homology with Q-SNARE complexes and mediates interaction with the plasma membrane complexes while secreted out from cells (Dismuke et al., 2012). Several studies have investigated the mechanism of MYOC multimerization. Full-length MYOC has been shown to interact with itself to form MYOC homodimers. This homodimerization is mediated majorly by the residues in the LZ domain and the CC region and is independent of the OLF domain (Fautsch and Johnson, 2001; Wentz-Hunter et al., 2002). MYOC was also observed to form higher-order complexes (Nguyen et al., 1998) through disulfide crosslinking (Gobeil et al., 2004). In AH, MYOC presents as a multimeric large complex with bands ranging from 120 to 180 kDa (Gobeil et al., 2004). Although the full-length MYOC crystal structure remains unresolved, the C-terminal OLF domain crystal structure indicates it comprises a five-bladed β-propeller featuring a metal binding center in its highly conserved interior region (Donegan et al., 2015). Furthermore, the N-terminal CC region exhibits a Y-shaped parallel dimer-of-dimer structure. Additionally, the crystal structure of the mouse myocilin LZ domain shows a dimer cap formed through C-terminal disulfide bonds, which are typical of a canonical leucine zipper (Hill et al., 2017). Altogether, the full-length MYOC forms a Y-shaped dimer-of-dimers consisting of an N-terminal tetrameric stalk from the CC/LZ region, connected to the two C-terminal OLF domains.

### Pathogenic MYOC variants in glaucoma

Mutations in the *MYOC* gene locus were the first glaucoma locus identified with a strong genetic link to POAG. *MYOC* genetic variants are estimated to account for about 4% of adult-onset POAG and >10% of autosomal dominant JOAG (Fingert et al., 1999; Aldred et al., 2004). Nearly 100 pathogenic variants in the *MYOC* gene have been found in a subset of patients with POAG (Fingert et al., 2002; Fingert et al., 1999), most of which cluster in exon 3, encoding the OLF domain. Recent studies also emphasize the accumulation of somatic *MYOC* mutations related to age in the onset of glaucoma (Sazhnyev et al., 2024). However, the observed *MYOC* gene variants in healthy control populations suggest that not all variants are pathogenic, but some are benign. While the physiological function of WT MYOC remains unclear, several mouse studies employing genetic strategies that either overexpress or disrupt *Myoc* gene expression did not lead to increased IOP or result in glaucoma (Kim et al., 2001; Gould et al., 2004; Gould et al., 2006). Also, haploinsufficiency of MYOC in patients due to either homozygous R46X truncation (Lam et al., 2000) or hemizygous interstitial deletion on 1q24-q26 does not cause glaucoma (Wiggs and Vollrath, 2001). Thus, the *MYOC* variants associated with glaucoma result from a toxic gain of function due to its misfolding (Zhou and Vollrath, 1999). The entire list of MYOC variants derived from extensive genome-sequencing projects can be found in the Genome Aggregation Database (gnomAD, https://gnomad.broadinstitute.org). Surprisingly, the MYOC β-propeller OLF domain accounts for over 90% of the reported missense and nonsense genetic variants. Based on various clinical and biochemical aspects of MYOC variants, Sclesi et al. categorize the pathogenicity of MYOC variants into three groups: pathogenic, likely pathogenic, and benign. The list of pathogenic variants includes C245Y, G246R, G252R, R272G, E323K, G364V, G367R, P370L, T377M, T377K, D380A, K423E, V426F, C433R, Y437H, I477N, I477S, Y479H, N480K, P481L, P481T, I499F, I499S, and S502P. The list of likely pathogenic variants includes V251A, P254R, L255P, P274R, Q337R, S341P, A363T, F369L, Y371D, T377R, D380H, D384G, E385K, T438I, N450Y, and L486F. Finally, the list of benign MYOC variants includes T293K, V329M, E352K, T353I, K398R, A445V, and K500R (Scelsi et al., 2021). Several studies showed that the disease-causing *MYOC* variants cause protein misfolding, amyloid-like aggregation, ER stress, and impaired secretion (Joe et al., 2003; Gobeil et al., 2004; Liu and Vollrath, 2004; Saccuzzo et al., 2024; Scelsi et al., 2023). In the following section, we will concentrate exclusively on specific MYOC variants that exhibit protein folding defects.

### Impaired secretion of MYOC variants

Recent findings indicate that variants of MYOC, which misfold in the ER, are responsible for their harmful gain of function in POAG disease. MYOC secretion by the secretory TM cells is vital for extracellular matrix (ECM)-mediated cell adhesion. However, the disease-causing MYOC E300K, E323K, S341P, G364V, P370L, D380A, K423E, C433R, Y437H, N450Y, I477N missense, and Q368X nonsense variants impaired cellular secretion and were predominantly retained in the ER (Joe et al., 2003; Liu and Vollrath, 2004; Aroca-Aguilar et al., 2008; Zadoo et al., 2016; Yan et al., 2020; Zhou et al., 2022a; Scelsi et al., 2023; Yan et al., 2024). In certain instances, mutant MYOC exhibits a dominant-negative effect by oligomerizing with WT, impacting WT MYOC secretion (Gobeil et al., 2004). Also, the MYOC levels were reduced in the POAG patients’ AH (Jacobson et al., 2001). Thus, the MYOC variants are misfolded, fail to pass the ER quality control mechanisms, and are retained in the ER, as evidenced by their prolonged interactions with ER chaperones such as BIP, PDI, CRT, and ERP57 (Joe et al., 2003; Scelsi et al., 2023; Liu and Vollrath, 2004). In some cases, the mutations caused the misfolding of the protein by hindering its Ca^2+^ binding ability (D380A) or leading to aberrant disulfide bond formation (C433R) due to insufficient cysteine or altered protein stability and aggregation (E300K, G364V, Q368X, P370L, D380A, K423E, Y437H).

The lack of secretion led to the accumulation of misfolded MYOC variants in the ER of TM cells, causing ER stress and triggering UPR pathway activation. Notably, the WT MYOC also inhibited secretion under conditions of ER Ca^2+^ depletion that induce ER stress (Saccuzzo et al., 2022). Studies involving the expression of MYOC S341P, Q368X, P370L, Y437H, N450Y, and I477N variants have demonstrated the upregulation of the ER stress marker BIP (Joe and Tomarev, 2010; Yan et al., 2024; Yan et al., 2020), along with activation of the UPR-PERK pathway, which coincided with an increase in the expression of ATF4 and CHOP (Yam et al., 2007a; Wang et al., 2007; Peters et al., 2015). Chronic levels of UPR activation occur because the ERAD pathway cannot effectively degrade MYOC variants (Qiu et al., 2014; Liu and Vollrath, 2004). Although the MYOC P370L and I477N variants are tagged for degradation through ubiquitination, their breakdown via the ERAD proteasome system fails due to mutant-specific prolonged interactions with GRP94 (Suntharalingam et al., 2012). Furthermore, the increase in ER stress and the activation of the PERK-ATF4-CHOP mediated UPR are correlated with the pathogenesis of glaucoma in multiple MYOC-dependent open-angle glaucoma mouse models expressing S341P, P370L, and Y437H variants. The elevated IOP, RGC death, and axon degeneration in these mouse models illustrate the molecular role of ER stress in glaucoma (Yan et al., 2024; Cheng et al., 2023; Kasetti et al., 2016; McDowell et al., 2012). Although the degree of UPR activation varied among the variants—from responses to oxidative stress to chronic activation of pro-apoptotic factors (Joe and Tomarev, 2010)—this finding aligns with previous studies indicating the crucial role of ATF4 expression results in dysfunction in TM cells, elevated IOP, and neurodegeneration (Peters et al., 2015; Kasetti et al., 2020).

### Aggregation and autophagic degradation of MYOC variants

Defects in protein folding within the ER and failure of ERAD to degrade misfolded proteins can lead to protein aggregation. Accumulating evidence suggests that pathogenic E300K, E323K, G364V, Q368X, P370L, D380A, K423E, and Y437H MYOC variants are sequestered in detergent-insoluble fractions, indicating a propensity to form intracellular aggregates instead of being secreted (Zhou and Vollrath, 1999; Liu and Vollrath, 2004; Yam et al., 2007a; Kasetti et al., 2021; Scelsi et al., 2023). The pathogenic MYOC variants tend to heterodimerize or hetero oligomerize with WT MYOC, forming inclusion bodies that sequester WT MYOC from extracellular secretion and affect TM function (Gobeil et al., 2004; Yam et al., 2007a). Similarly, in various cells, the *in vitro* expression of E323K, G364V, P370L, D380A, and K423E MYOC variants demonstrated juxtanuclear aggregates (Liu and Vollrath, 2004). Similar findings were observed when the OLF domain-specific expression of P370L-OLF and Y437H-OLF variants as MBP fusion proteins in *E. coli* revealed their presence in the void volume, suggesting the formation of high molecular weight species (Burns et al., 2010; Orwig et al., 2012). These high-molecular-weight OLF domain aggregates exhibited morphological characteristics similar to protofibril oligomers and amyloid fibrils. When expressed in CHO cells, these amyloid-like aggregates were also noted in the context of full-length P370L MYOC protein (Orwig et al., 2012). Further, these aggregated variants (G364V, P370L, K423E, Y437H, I477N) that are retained in the ER cause cytotoxicity to the TM cells (Joe et al., 2003; Liu and Vollrath, 2004). These studies indicate that MYOC variants with folding defects that tend to aggregate are associated with a severe glaucoma phenotype. In contrast, variants displaying greater solubility are linked to a milder glaucoma phenotype (Scelsi et al., 2021).

The Ca^2+^ binding OLF domain of MYOC is responsible for pathogenic amyloid-like aggregation (Orwig et al., 2012). The WT MYOC also aggregates under ER stress conditions in a Ca^2+^-dependent manner. Depleting cellular Ca^2+^ causes WT MYOC to accumulate inside the ER, becoming less soluble and aggregating like amyloid (Saccuzzo et al., 2022; Saccuzzo et al., 2024), suggesting that the aggregated WT MYOC may intensify ER stress (Saccuzzo et al., 2023). Although the WT OLF domain folds properly, conditions such as agitation, acidic pH, high temperature, or mutations encourage amyloid-like aggregation akin to disease-causing variants (Saccuzzo et al., 2024; Orwig et al., 2012). Mechanistically, the treatment of urea created a partially folded state of β-propeller, with the unfolding of WT OLF resulting from the central residues. At the same time, the exterior blades provide resistance to unfolding (Saccuzzo et al., 2024). The morphology of WT MYOC fibril formation varies between straight and circular fibrils based on the type of stress. Differences in fibril formation were also noted among the pathogenic variants. The highly pathogenic I499F variant displayed straight fibrils with an increase in aggregation rate, whereas the moderately pathogenic D380A variant showed circular fibrils (Hill et al., 2014; Saccuzzo et al., 2024; Burns et al., 2011). Compared to WT OLF, the variants D380A and I499F adopt unique partially folded structures, partly due to their differences in Ca^2+^ binding, accounting for the variations in fibril formation (Saccuzzo et al., 2024).

**FIGURE 2:**
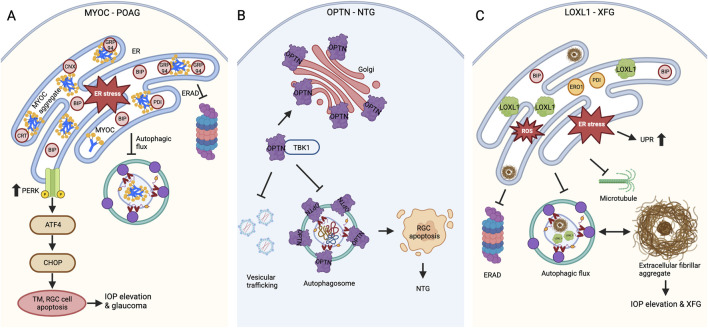
Defects in protein folding and proteostasis in glaucoma **(A)** MYOC variants causing POAG. MYOC mutations lead to amyloid-like protein aggregation, while insufficient secretion results in ER stress and activates the PERK-ATF4-CHOP UPR pathway. Further, the inadequate ERAD and defects in autophagic flux cause TM and RGC cell death through apoptosis, which leads to IOP elevation and glaucomatous neurodegeneration. **(B)** OPTN variants causing NTG. OPTN mutations cause altered localization with increased foci formation around the Golgi apparatus. The enhanced interaction of E50K OPTN with TBK1 affects RAB8-mediated vesicular trafficking and autophagosome formation, which ultimately contributes to RGC apoptosis and glaucomatous neurodegeneration. **(C)** LOXL1 polymorphisms contribute to the risk of XFG. In XFG, the exfoliative fibrillar aggregate material builds up because of the loss of cellular proteostasis, resulting in ER stress, UPR, defective ERAD, and autophagy. The pathogenic LOXL1 variant expression also caused aggregation, ER-oxidative stress, and defective autophagy.

When the ER becomes overloaded with misfolded protein aggregates and the ERAD system fails, autophagy is triggered to serve a cytoprotective role mechanism. Some of the misfolded S341P, P370L, Y437H, N450Y, and I477N MYOC variants are reported to be degraded through the autophagy pathway (Yan et al., 2024; Qiu et al., 2014; Suntharalingam et al., 2012; Kasetti et al., 2021; Yan et al., 2022). However, whether these aggregated ER-retained MYOC variants are specifically degraded through ER-phagy remains unexplored. The induction of ER stress and UPR activation, combined with the extended interaction of misfolded variants with ER chaperones, indicates that the chaperones’ attempts at refolding were unsuccessful. This failure, along with fibril formation, complicates the fate of these variants, leading to their degradation via the autophagy pathway and causing TM and RGC dysfunction. The interaction between MYOC amyloids and the GRP94 chaperone directs the aggregates toward ERAD while obstructing the ERAD pathway, resulting in inefficient degradation. Interestingly, preventing the GRP94 interaction allowed the MYOC amyloids (P370L, I477N) to degrade through autophagy efficiently (Suntharalingam et al., 2012). However, recent studies indicate that while misfolded variants are targeted for autophagy, the altered autophagic flux in S341P and Y437H variants has led to diminished autophagic clearance, which accounts for chronic ER stress (Yan et al., 2024; Kasetti et al., 2021). Crucially, reducing CHOP or enhancing autophagic flux facilitated the degradation of Y437H, promoted cellular homeostasis, and lowered IOP levels (Kasetti et al., 2021). Thus, MYOC variants cause a toxic gain-of-function due to misfolding, leading to the formation of amyloid fibrils that induce ER stress and activate the PERK-ATF4-CHOP pathway while disrupting autophagic flux (Figure 2A) and (Table 1).

**TABLE 1:** Pathogenic mechanism and potential drug target for glaucoma mutants.

Disease	Mutation	Model system	Pathogenic mechanism	Potential drug target	References
POAG	MYOC E300K	Cell lines	Impaired secretion, aggregation		Scelsi et al. (2023), Zadoo et al. (2016)
POAG	MYOC E323K	Cell lines	Impaired secretion, insoluble, aggregation		Aroca-Aguilar et al. (2008), Liu and Vollrath (2004), Zhou and Vollrath (1999)
POAG	MYOC S341P	TM cells, mouse model	ER stress, autophagy dysfunction, TM, RGC dysfunction	4-PBA	Yan et al. (2024)
POAG	MYOC G364V	Cell lines, TM cells	Impaired secretion, aggregation, TM cytotoxicity		Liu and Vollrath (2004), Joe et al. (2003), Kasetti et al. (2021)
POAG	MYOC Q368X	Cell lines, TM cells	Impaired secretion, heterooligomerize with WT, ER stress, UPR, defective proteasomal degradation, TM dysfunction		Aroca-Aguilar et al. (2008), Joe et al. (2003), Sohn et al. (2002), Zhou and Vollrath (1999), Gobeil et al. (2004), Qiu et al. (2014)
POAG	MYOC P370L	Cell lines, TM cells, mouse model	Impaired secretion, insoluble, amyloid-like aggregation, ER stress, UPR, impaired degradation, oxidative stress, mitochondrial dysfunction, TM and RGC dysfunction	Recombinant anti-OLF antibodies	Liu and Vollrath (2004), Aroca-Aguilar et al. (2008), Zhou et al. (2022a), Scelsi et al. (2023), Joe and Tomarev (2010), Yam et al. (2007a), Suntharalingam et al. (2012), Cheng et al. (2023), Orwig et al. (2012), Qiu et al. (2014), Wang et al. (2007), He et al. (2009), Ma et al. (2025)
POAG	MYOC D380A	Cell lines	Impaired secretion, insoluble, circular fibril aggregation		Liu and Vollrath (2004), Aroca-Aguilar et al. (2008), Zhou and Vollrath (1999), Saccuzzo et al. (2024)
POAG	MYOC K423E	Cell lines	Impaired secretion, insoluble, heterooligomerize with WT, aggregation, TM cytotoxicity		Liu and Vollrath (2004), Joe et al. (2003), Zhou et al. (2022a), Zhou and Vollrath (1999), Gobeil et al. (2004)
POAG	MYOC C433R	Cell lines	Impaired secretion, impaired disulfide bond formation		Zhou et al. (2022a), Donegan et al. (2015)
POAG	MYOC Y437H	Cell lines, mouse model	Impaired secretion, insoluble, aggregation, ER stress, oxidative stress, UPR, autophagy dysfunction, TM cytotoxicity, RGC death	4-PBA, Torin-2, Tat-Beclin-1, 4-Br-BnIm, CRISPR-Cas9-based genome editing, TM stem cell transplantation	Liu and Vollrath (2004), Joe et al. (2003), Zadoo et al. (2016), Zhou et al. (2022a), Joe and Tomarev (2010), Zhou and Vollrath (1999), Zode et al. (2011), Kasetti et al. (2021), Stothert et al. (2014), Kasetti et al. (2016), Jain et al. (2017), Patil et al. (2024), Xiong et al. (2021), Zhu et al. (2016)
POAG	MYOC N450Y	TM cells, mouse model	Impaired secretion, ER stress, autophagy, TM and RGC death	4-PBA	Yan et al. (2020), Yan et al. (2022)
POAG	MYOC I477N	Cell lines	Impaired secretion, insoluble, ER stress, oxidative stress, defective ERAD, TM cytotoxicity	4-Br-BnIm, Recombinant anti-OLF antibodies	Zadoo et al. (2016), Joe et al. (2003), Zhou et al. (2022a), Joe and Tomarev (2010), Zhou and Vollrath (1999), Suntharalingam et al. (2012), Stothert et al. (2014), Ma et al. (2025)
POAG	MYOC I499F	Purified protein	Partially insoluble, fibrillar aggregation		Burns et al. (2011), Scelsi et al. (2023)
NTG	OPTN E50K	Cell lines, hPSCs, mouse model	Insoluble, aggregation, defective autophagy, defective mitochondrial dynamics, increased ROS, RGC apoptosis	Amlexanox, BX795	Gao et al. (2014), Chalasani et al. (2014), VanderWall et al. (2020), Zhang et al. (2021a), Shim et al. (2016), Chalasani et al. (2007), Minegishi et al. (2013), Minegishi et al. (2016), Surma et al. (2023)
NTG	OPTN M98K	Cell lines	ER stress, UPR activation, increased autophagy, RGC cell death	BX795	Sayyad et al. (2023), Sirohi et al. (2013), Sirohi et al. (2015)
XFG	LOXL1 R141L, G153D	Cell lines	Charge alteration, aggregation, ER-oxidative stress, defective autophagy		Sharma et al. (2016), Bernstein et al. (2019), Bernstein et al. (2018), Venkatesan et al. (2023)

### Optineurin

Optic neuropathy-inducing protein (Optineurin, OPTN) is a cytoplasmic protein encoded by the *OPTN* gene. OPTN has several functional domains, including three CC domains, a LZD, an LIR motif, a ubiquitin-binding domain (UBD), and a C-terminal zinc finger domain. OPTN predominantly localizes in the cytoplasm and has multi-functional properties involved in vesicular trafficking, autophagic clearance of protein aggregates and defective mitochondria, and the inflammatory response. OPTN, as an adapter protein, executes its diverse function by interacting with its binding proteins, including RAB8, myosin VI, TFR, TBK1, huntingtin, transcription factor IIIA, and inflammatory and interferon signaling (Ying and Yue, 2012). The aggregation of OPTN protein has been implicated in ALS and other neurodegenerative diseases (Osawa et al., 2011).

OPTN plays a key role in Golgi-mediated vesicular trafficking. When overexpressed, the OPTN forms foci around the Golgi apparatus, leading to its fragmentation and apoptosis (Park et al., 2006). These OPTN foci are also induced in response to viral RNA, which leads to the dampening of pro-inflammatory immune responses (O'Loughlin et al., 2020). In its native form, OPTN appears as homo-hexamers. The homo-hexamerization seems to be the functional form, as indicated by the assembly of super-complexes with vesicular trafficking proteins like RAB8, myosin VI, and TFR (Ying et al., 2010). OPTN is a soluble macro autophagy receptor that binds ubiquitin and plays a crucial role in degrading protein aggregates, faulty mitochondria, and intracellular bacterial pathogens through autophagy. It has demonstrated its ability to facilitate the maturation of autophagosomes during the degradation process of various cargoes, including the degradation of α-synuclein in Parkinson’s disease (Wise and Cannon, 2016). The mechanism by which OPTN regulates autophagy involves the critical binding of TBK1 to the N-terminal CC domain of OPTN, which is essential in controlling the latter’s autophagy process. When OPTN identifies the ubiquitinated cargo, TBK1 phosphorylates the S177 residue of the OPTN LIR motif, enhancing its binding affinity for LC3. Additionally, phosphorylation of the S473 residue in the UBD domain further strengthens OPTN’s connection to ubiquitinated cargoes. Consequently, TBK1’s dual phosphorylation facilitates OPTN in sequestering cargoes into autophagosomes (Heo et al., 2015; Wild et al., 2011). Likewise, OPTN is recruited to the damaged, ubiquitinated mitochondria mediated by the PINK1-Parkin pathway and transports them to autophagosomes for degradation (Heo et al., 2015). Also, recent studies show that the C-terminal domain of OPTN regulates the axonal transport of mitochondria by interacting with microtubules (Liu et al., 2025). While OPTN resides in the cytosol, any issues with its expression can disrupt ER and mitochondrial homeostasis, likely by influencing autophagy regulation. The knockdown of OPTN led to heightened ER stress, activation of the PERK-ATF4 UPR pathway, increased reactive oxygen species (ROS) levels, enhanced chaperone-mediated autophagy, and impaired macro autophagy (Ali et al., 2019). Beyond its role in vesicular trafficking and autophagy, OPTN regulates inflammatory signaling pathways to prevent chronic inflammation. OPTN shares 53% sequence homology with NF-κB essential modulator and acts as a negative regulator of TNFα-induced NF-κB activation (Slowicka and van Loo, 2018). Therefore, mutations in OPTN disrupt its biological function and could ultimately result in disease.

### Pathogenic OPTN variants in glaucoma

Mutations in OPTN are associated with NTG and ALS, a fatal motor neuron disease. Missense mutations in OPTN, such as H26D, E50K, and M98K, along with H486R and E322K, are associated with NTG, which leads to RGC damage without an increase in IOP.

The OPTN E50K (RGC) mutation is the most common mutation linked to NTG. While the WT OPTN is soluble and forms homo-oligomers to perform its function during normal physiological conditions, the overexpression of E50K OPTN causes the protein to misfold and accumulate more in the insoluble fraction, forming improper higher-order oligomeric complexes. Further studies revealed that the OPTN mutations induce a novel trimer and oligomers via the UBD, which resembles the complexes observed during oxidative stress in WT OPTN (Gao et al., 2014). This could be partly due to the change of amino acid charge in the mutant, which may have led to enhanced interaction of E50K OPTN oligomers with TBK1 (Ying et al., 2010). The insoluble higher-order complex formation of E50K OPTN caused its altered cellular localization and cell death. The foci formation is increased around the Golgi apparatus and ER organelles in the E50K over-expressing cells, patient-derived induced pluripotent cells, and mouse models (Minegishi et al., 2013; Park et al., 2006). Furthermore, the modified localization also hindered its interaction with RAB8, a regulator of vesicular trafficking, and impacted TFR complex recycling (Vaibhava et al., 2012; Chalasani et al., 2014).

E50K OPTN overexpression causes defects in proteasomal and autophagy-mediated degradation, ultimately triggering apoptosis in RGCs. While both WT and E50K mutant overexpression reduce proteasomal activity, E50K overexpression uniquely affects autophagic flux, resulting in decreased autophagosome formation. This leads to the accumulation of misfolded proteins and organelles, causing toxicity (Chalasani et al., 2014; Shen et al., 2011). The expression of OPTN E50K in a human pluripotent stem cell model exhibited autophagy defects alongside neurodegenerative issues, including neurite retraction and hyperexcitability (VanderWall et al., 2020). In addition to impaired autophagic degradation, E50K OPTN overexpression also affected the degradation of TDP43, a protein involved in mRNA biogenesis with implications for ALS. E50K, but not WT OPTN interaction with TDP43, impairs its autophagic degradation, accumulating TDP43 in the cytosol of the retina and contributing to proteinopathy (Zhang et al., 2021a). Moreover, defective autophagy in E50K OPTN cells is causative for increased foci formation and apoptosis. However, these defects are reversed by inducing autophagy with rapamycin (Shen et al., 2011; Chalasani et al., 2014). The defects in mitochondrial dynamics also contribute to the degeneration of RGCs in E50K OPTN (Shim et al., 2016). The mitochondrial defects are thoroughly discussed in the later sections of this review. Thus, the E50K mutation in OPTN causes a toxic gain-of-function due to altered interactions, improper localization, and impaired autophagy, ultimately leading to the death of photoreceptor cells in NTG pathogenesis (Figure 2B).

Unlike E50K, the M98K OPTN polymorphism is associated with NTG at higher rates in Asian and African populations but not in Caucasian or Hispanic groups (Ayala-Lugo et al., 2007; Rezaie et al., 2002). This suggests that the M98K OPTN polymorphism alone is not a causative factor for glaucoma pathogenesis. Although research on the impact of the M98K OPTN mutation on solubility and oligomerization is limited, recent evidence suggests that this variant leads to the death of RGCs because of increased autophagy. The expression of M98K OPTN increases the number of autophagosomes due to the involvement of ATG5. Additionally, cells overexpressing M98K OPTN displayed enhanced UPR activations with increased IRE1α, PERK, and ATF6 expressions. Retinal cells with M98K OPTN become more sensitive to TNFα and tunicamycin, an ER stress agent. This is due to its modulation of ER stress response signaling pathways, potentially leading to increased autophagy and cell death through CHOP-mediated caspase activation (Sayyad et al., 2023). The mechanism of cell death is also reasoned by the stronger interaction of M98K with RAB12 than WT OPTN. RAB12, found in autophagosomes, plays a role in regulating vesicular trafficking. The enhanced activation of RAB12 by M98K OPTN causes abnormal lysosomal degradation of TFR and causes cell death (Sirohi et al., 2013). However, the degradation of TFR is also regulated by OPTN’s binding partner, TBK1. While the association of TBK1 is reduced with M98K OPTN, the increase in autophagy was due to the prolonged phosphorylation of S177 of M98K OPTN by TBK1 (Sirohi et al., 2015). Thus, the M98K OPTN polymorphism causes disease through a gain-of-function mechanism that enhances autophagic flux, leading to inflammasome activation and cell death (Table 1).

### Lysyl oxidase-like 1

Lysyl oxidase-like 1 (LOXL1) is part of the LOX family of enzymes (LOX, LOXL1-4). These enzymes are involved in the covalent cross-linking of lysine residues in elastin and collagen fibrils within the ECM, facilitating tissue integrity. While LOX and LOXL1 contribute to maintaining ECM integrity, LOXL2-4 are implicated in fibrosis and various cancers (Rodriguez-Pascual and Rosell-Garcia, 2018). The LOXL1 protein consists of two domains, with the C-terminal copper binding-dependent catalytic domain being highly conserved among the LOX protein family. The N-terminal domain varies within the lysyl oxidases, and the proline-rich N-terminal domain of LOXL1 is predicted to have an intrinsically disordered region (IDR) (Bernstein et al., 2019). While LOXL1 is encoded as an inactive pre-pro-enzyme, it becomes catalytically active after the N-terminal cleavage of its pro-region by BMP-1 protease in cell supernatants. This enables the catalytically active LOXL1 protein to cross-link its client proteins, such as tropoelastin monomers, through its oxidative deamination activity (Thomassin et al., 2005). Although the C-terminal domain is responsible for this cross-linking activity, the N-terminal IDR region plays a critical role in elastogenesis by interacting with fibulin-5 to promote efficient polymer formation (Liu et al., 2004).

### Pathogenic LOXL1 variants in glaucoma

XFG is a systemic disease associated with aging, marked by abnormal ocular elastosis and the accumulation of extracellular fibrillar aggregates in the AH outflow pathway, resulting in elevated IOP. The glaucomatous degeneration resulting from exfoliation material includes elastic fiber components like elastin, fibrillin-1, fibulins, latent TGF-β binding proteins, and LOXL1 (De Maria et al., 2021; Zenkel and Schlotzer-Schrehardt, 2014), which is the recognized cause of secondary open-angle glaucoma, XFG. GWAS studies have identified polymorphisms in the *LOXL1* gene as the major risk factor for the development of XFG. The common nonsynonymous LOXL1 polymorphisms R141L and G153D reside in exon 1, which encodes the N-terminal IDR domain (Aung et al., 2017). Although these LOXL1 polymorphisms are reversed in some populations, they remain the primary XFG-associated risk factors reported across multiple populations worldwide (Li et al., 2021). While the LOXL1 crystal structure remains unresolved, *in silico* analyses have revealed that substitutions in LOXL1 from both R141L and G153D resulted in charge alterations. These changes may impact the local electrostatic potential of the protein, potentially affecting its interaction partners (Sharma et al., 2016). Expression of R141L and G153D LOXL1 variants do not impact cellular secretion and extracellular accumulation (Aung et al., 2017; Sharma et al., 2016). Moreover, the variants do not influence the amine oxidase activity of LOXL1 when using recombinant proteins (Kim and Kim, 2012). However, the BMP-1 protease processing of LOXL1 was altered when over-expressed as an L141_G153 variant, but its extracellular accumulation was not altered (Sharma et al., 2016). Thus, LOXL1 variants in XFG do not primarily affect its localization and function.

Although knowledge about the cellular biological effects of LOXL1 variants on protein folding and aggregation is limited, over-expression of the N-terminal IDR domain alone has shown punctate aggregates. These aggregates seem more pronounced under serum-free conditions and stained positive for the protein aggregation dye Proteostat, indicating that LOXL1 may misfold during cellular stress. The aggregation might stem from the two IDR domain disordered peak residues 28 and 125 since their removals decreased the aggregation. Both high-risk LOXL1 variants coincide with the IDR peaks, indicating that LOXL1 has a higher potential for aggregation in XFG, aligning with its presence in the exfoliation material (Bernstein et al., 2019). Consistent with the aggregation, overexpression of LOXL1 in our lab induced ER stress, as evidenced by increased BIP expression. However, the ER stress did not activate the UPR; instead, it triggered the activation of the oxidative folding pathway, accompanied by increased expressions of PDI and ERO1α (Venkatesan et al., 2023). This signifies the increase of LOXL1 occurs during profibrotic growth factor TGF-β and under oxidative stress conditions.

XFG is an aging-related disease, and understanding its pathogenesis is hindered by the lack of an animal model (Anderson et al., 2018). Although *LOXL1* is a major risk variant in XFG, knocking down *Loxl1* in mouse models did not produce an XFG phenotype (Wareham et al., 2022; Wiggs et al., 2014). Lens tissue-specific overexpression of mouse *Loxl1* showed an increase in IOP at 1 month but no differences in 2-month-old transgenic animals. Intriguingly, overexpression enlarged rough ER cisterns filled with electron-dense Loxl1 aggregates measuring 20–30 nm in diameter, demonstrating that LOXL1 is an aggregation-prone protein influencing the fibrotic process (Zadravec et al., 2019). Emerging evidence also indicates that LOXL1 is degraded through the autophagy pathway, where the autophagic flux is altered in the primary Tenon fibroblast (TF) cells explanted from XFG patient Tenon tissues (Table 1) (Bernstein et al., 2018). Furthermore, these primary TF cells exhibited signs typically associated with protein misfolding, leading to neurodegenerative diseases. These signs include defects in autophagosome clearance rates, microtubule organization, and mitochondrial depolarization (Want et al., 2016; Wolosin et al., 2018). The decreased proteasome activity and impaired UPR in the lens capsule of XFG patients also contribute to the accumulation of misfolded proteins and impact autophagy (Hayat et al., 2019). Thus, available data supports that the XFG is an aggregopathy disease, where the proteostasis imbalance caused the accumulation of misfolded aggregates and contributed to the buildup of exfoliation material (Figure 2C). Future research will explore how these aggregates are formed and organized outside of cells and identify mechanisms to enhance proteostasis and prevent increases in IOP.

### Mitochondrial dysfunction in glaucoma

**FIGURE 3:**
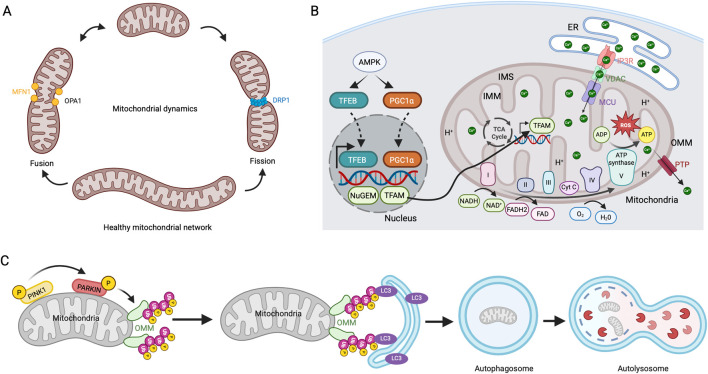
Mitochondrial dynamics, biogenesis, respiration, and mitophagy **(A)** Mitochondrial fission and fusion mechanisms. Mitochondria continuously undergo fission and fusion cycles. DRP1 regulates the fission process, while MFN1 and OPA1 drive its fusion. **(B)** Mitochondrial biogenesis and respiration. Involvement of, ETC, in ATP production. ER releases Ca^2+^ through the IP3R receptor, which is imported into the mitochondrial matrix via MCU and released through PTP. **(C)** PINK1/PARKIN mediated mitophagy pathway. In dysfunctional mitochondria, stabilizing PINK1 in the OMM leads to the activation and recruitment of PARKIN. This action initiates the polyubiquitination of OMM proteins, recruitment of LC3-coated autophagosomes, and degradation of mitochondria in autolysosomes.

Mitochondria are dynamic organelles with a double membrane that is a major source of cellular ATP. Although mitochondria possess their own genome (mtDNA) that encodes genes related to oxidative phosphorylation (OXPHOS), they largely depend on nuclear DNA for other mitochondrial proteins, which are imported by the translocase of the outer mitochondrial membrane (OMM) and inner mitochondrial membrane (IMM) TOM/TIM machinery. Mitochondria continually alter their shape through fission and fusion processes, crucial for sustaining mitochondrial function, energy generation, and cellular homeostasis. The dynamin-related super-family GTPase proteins regulate the fission-fusion events, with DRP1 controlling mitochondrial fission (Smirnova et al., 2001). Mitochondrial fusion is regulated by OPA1 in IMM and MFN1, and 2 in the OMM (Santel and Fuller, 2001) (Figure 3A). Mitochondrial ATP production occurs in the cristae of the IMM, controlled by the electron transport chain (ETC) complexes (I-IV) and ATP synthase (complex V) that shuttle electrons from the oxidation of nutrients in the form of NADH and FADH_2_ to molecular oxygen, generating protonmotive force (Kuhlbrandt, 2015) (Figure 3B). During this process, ROS produced as a by-product in the IMM are effectively neutralized by an antioxidant defense system in healthy cells. However, the inability to remove the ROS directly impacts mtDNA and the, ETC, leading to the loss of membrane potential, a decrease in bioenergetic capacity, and apoptosis in age-related pathologies (Balaban et al., 2005). Mitochondrial calcium (MitCa^2+^) homeostasis plays a crucial role in the above-mentioned processes, as it alters the organelle’s functions and mitochondrial dynamics. The MitCa^2+^ is received via the ER through the mitochondrial calcium uniporter (MCU) complex, and the excess Ca^2+^ influx is effectively removed through the permeability transition pore (PTP) to prevent ROS elevation, cytochrome c release, and apoptosis (Pinton et al., 2008). Mitochondria are constantly exposed to cellular stress and ROS. To maintain homeostasis, these organelles have developed a mechanism to eliminate dysfunctional mitochondria through mitophagy, a selective autophagic process. The most well-studied mechanism for mitophagy is the canonical PINK1/Parkin pathway. In the functional mitochondria, PINK1 in OMM is imported into the IMM, rapidly cleaved, and degraded by mitoprotease. However, PINK1 is stabilized by auto-phosphorylation at OMM in the damaged mitochondria. PINK1 recruits an E3 ubiquitin ligase, Parkin, to polyubiquitinate OMM proteins further recognized by the autophagy receptors and direct the mitochondria for autophagosome formation and degradation (Figure 3C) (Lazarou et al., 2015).

Emerging evidence indicates mitochondrial dysfunction in glaucoma patients and various animal models of glaucoma involving the TM in the anterior chamber and RGCs in the posterior eye segment. Initial studies showed that mitochondrial respiratory activity was reduced in the lymphocytes of POAG patients with mtDNA changes in complexes I, III, IV, and V (Abu-Amero et al., 2006). The mitochondrial bioenergetics, which is considered an important measure to assess pathogenicity in different mitochondrial diseases, is impaired in POAG patient-derived ocular TF cells and peripheral blood mononuclear cells (Vallabh et al., 2022; Petriti et al., 2024). Also, the TM cells from the outflow flow pathway of POAG patients showed increased ROS accumulation, decreased ATP levels, and lower mitochondrial membrane potential (MMPT). These defects are exacerbated when the mitochondria are inhibited with the complex I inhibitor Rotenone, leading to TM cell apoptosis (He et al., 2008b). The increased accumulation of ROS in primary cells is consistent with increased oxidative mtDNA damage in the TM cells from the POAG and XFG glaucoma patients (Izzotti et al., 2003), highlighting the role of oxidative stress in glaucoma. Additionally, genetic studies reporting SNPs in nuclear and mtDNA genes that encode mitochondrial proteins explain the susceptibility to glaucoma associated with mitochondria (Lascaratos et al., 2012). Similar to mitochondrial dysfunction in the anterior eye chamber, RGCs from patients with POAG demonstrated degeneration due to programmed cell death (Kerrigan et al., 1997). Further studies involving glaucoma donors’ lamina cribrosa cells revealed oxidative stress and mitochondrial dysfunction, as evidenced by impaired redox and Ca^2+^ homeostasis (McElnea et al., 2011). Recent studies also support these findings from POAG patients’ ONH measuring flavoprotein fluorescence. They show that mitochondrial dysfunction is caused by the oxidation of mitochondrial flavoproteins at the ONH rim due to glaucomatous damage (Zhou et al., 2022b). Thus, oxidative stress occurs in glaucoma, and mitochondrial dysfunction may be a cause or consequence of TM and RGC cell apoptosis.

### IOP and mitochondrial dysfunction in RGCs of glaucoma models

**FIGURE 4:**
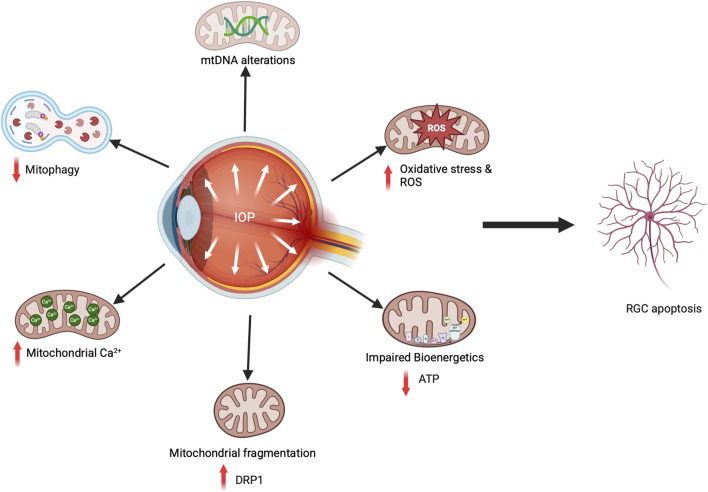
Mitochondrial dysfunction in glaucoma causes RGC death. In glaucomatous animal models, elevated IOP and oxidative stress result in mtDNA mutations, decreased ATP production, increased fission, enhanced mitochondrial Ca^2+^ transport from the ER, and reduced mitophagy flux, all contributing to RGC cell death.

Several glaucomatous animal models have been studied, enhancing our understanding of mitochondrial dysfunction related to glaucoma pathophysiology. Mitochondria are essential to the ONH, and mitochondrial dysfunction and RGC apoptosis are key features of glaucomatous optic neuropathy. Due to their phototransduction function, retinal neurons have the highest energy demands, making them susceptible to oxidative stress and mechanical stretch injuries caused by elevated IOP. In glaucoma, RGC apoptosis occurs due to various factors, including aging, elevated IOP, oxidative stress, and mitochondrial dysfunction (Chrysostomou et al., 2010). Studies with glaucoma animal models have clearly shown this association. In the D2 secondary glaucoma mouse model, elevated IOP leads to oxidative stress and mitochondrial defects, including reduced OXPHOS and ATP production, altered morphology of mitochondria and cristae, and impaired mitophagy (Coughlin et al., 2015). Similarly, the IOP increase in Wister rats showed decreased ATP production with increased ROS and extensive mtDNA damage, contributing to RGC loss (Wu et al., 2015). The IOP increase in OHT mouse models also revealed an unusual accumulation of mitochondria in the Soma and axons (Maddineni et al., 2020; Kaipa et al., 2025). Additional research indicated hydrostatic pressure exposure in RGC cells influences mitochondrial fission-fusion processes, resulting in mitochondrial fragmentation and decreased ATP production (Ju et al., 2007). This indicates that diminished energy availability for RGCs makes them susceptible to apoptosis and glaucomatous damage. Recent research has shed light on how mitochondrial dysfunction occurs in glaucoma, highlighting the role of the mitochondrial fission-fusion cycle in safeguarding RGCs (Ju et al., 2010; Kim et al., 2015; Ju et al., 2008). Kim et al. proposed a vicious cycle in which elevated IOP in D2 glaucomatous mice leads to oxidative stress. This oxidative stress triggers enhanced mitochondrial fission through DRP1, leading to mitochondrial fragmentation in RGCs. Inhibiting DRP1 or over-expressing OPA1 safeguards RGCs, indicating a significant mitochondria-dependent pathophysiological mechanism in glaucomatous neurodegeneration (Kim et al., 2015; Ju et al., 2010). Additionally, these models revealed the presence of inflammatory signaling in RGCs linked to mitochondrial dysfunction (Jassim et al., 2021), signifying the complexity of the disease. Thus, increasing IOP directly affects mitochondrial health and causes RGC cell death (Figure 4).

### Disease-causing mitochondrial variants in glaucoma

In addition to somatic and age-related mutations, mtDNA damage contributes to the development of various degenerative diseases, including glaucoma (Lascaratos et al., 2012). Twenty-two distinct transversion mutations in mitochondrial complex genes that impact mitochondrial bioenergetics were reported from peripheral blood leukocytes of POAG patients (Abu-Amero et al., 2006). Complex I mutations were also observed in the peripheral blood leukocytes of POAG patients from diverse ethnic backgrounds (Sundaresan et al., 2015), consistent with the impaired complex I-linked respiration (Lee et al., 2012). Studies from European populations have identified SNPs in the complex I genes, *MT-ND2*, *MT-ND4*, and the complex III gene, *MT-CYB* (Lo Faro et al., 2021; Singh et al., 2018). The common 4,977 bp mtDNA deletions were significantly elevated when analyzed in the POAG patients’ ocular tissue, specifically the TM (Izzotti et al., 2010). The development of next-generation sequencing (NGS) technologies confirmed the heteroplasmy in the mitochondrial complex genes associated with POAG and highlighted the importance of using ocular tissues for genetic studies (Vallabh et al., 2024).

While large-scale mtDNA deletions were uncommon in NTG, SNPs in mitochondrial complex I genes were noted (Piotrowska-Nowak et al., 2019). NGS studies of mtDNA identified genetic variants across 21 genes, with polymorphisms in *MT-ND2*, *MT-COX3*, and *MT-CYTB* showing increased frequency in the peripheral blood of NTG patients (Jeoung et al., 2014). Various groups have identified polymorphisms in nuclear DNA-encoded mitochondrial genes, including *OPA1*, *MFN1*, and *MFN2*, indicating an imbalance between mitochondrial fission and fusion in NTG (Lascaratos et al., 2012). While genetic studies increasingly report mitochondrial gene variants in NTG, research is lacking to understand the mechanisms of mitochondrial dysfunction caused by these variants in NTG pathogenesis. In XFG, GWAS studies conducted across various ethnic populations did not identify SNPs on mitochondrial genes. However, a 6.3-fold increased likelihood of mtDNA deletions was reported in the TM of XFG patients (Izzotti et al., 2011). Additionally, SNPs in complex I and II genes were observed in the Saudi Arabian population (Abu-Amero et al., 2008). The role of mitochondrial gene mutations in disease outcomes is a burgeoning field of study. Although expansive studies are not yet available, these studies collectively highlight the importance of mitochondrial genetic defects in POAG, NTG, and XFG pathogenesis.

In POAG, most reported mitochondrial gene polymorphisms occur in the mitochondrial complex I genes, directly impacting OXPHOS. The various IOP-inducing POAG glaucoma mouse models have also revealed diverse mitochondrial defects that contribute to RGC death. Although MYOC is not a mitochondrial protein, its mutations have been shown to affect mitochondrial function. The highly pathogenic MYOC P370L variant expression causes cellular apoptosis due to mutant MYOC aggregation in the ER, induces ER stress, and fails to clear via autophagy (Wang et al., 2007). In addition to protein folding defects, the P370L MYOC variant expression in human TM cells also caused mitochondrial dysfunction with decreased ATP production, lower MMPT, and higher ROS and cellular Ca^2+^ levels. Interestingly, while MYOC is a secreted protein, the overexpression of both WT and P370L MYOC variants led to the localization of MYOC in the mitochondria (He et al., 2009). This indicates that MYOC may have an unexplored role in regulating mitochondrial function.

While OPTN is not a mitochondrial protein, mutations in the *OPTN* gene in patients having NTG have been shown to cause mitochondrial dysfunction. OPTN is identified as one of the autophagy receptors and manages the PINK1/Parkin-mediated degradation of defective mitochondria through its UBD and LIR domains. After identifying its substrates, OPTN interacts with LC3 via the LIR domain to regulate autophagosome maturation. The mechanism of OPTN in regulating mitophagy has recently been clarified. OPTN is recruited to the OMM of depolarized mitochondria through a Parkin-dependent mechanism, with its UBN domain providing stabilization. The TBK1-dependent phosphorylation of OPTN also facilitates the recruitment of OPTN to the cargo site and boosts its binding to the ubiquitinated mitochondria (Heo et al., 2015; Richter et al., 2016). However, in ALS disease, the failure of ALS OPTN mutant recruitment impairs autophagosome maturation and disrupts mitophagy, accumulating misfolded mitochondrial proteins, ultimately contributing to disease pathogenesis (Wong and Holzbaur, 2014). Intriguingly, none of the OPTN glaucoma variants influenced OPTN recruitment for mitophagy, suggesting a distinct disease mechanism (Chernyshova et al., 2019).

The expression of E50K OPTN led to defective autophagy and RGC cell death. It also increased ROS levels through an unknown mechanism. Notably, antioxidant treatment prevented RGC death, indicating that oxidative stress plays a role in disease development (Chalasani et al., 2007). Nevertheless, the role of OPTN pathological variants in regulating mitochondrial biogenesis remained unclear. Recently, the RGC death in E50K OPTN NTG has been demonstrated due to mitochondrial-mediated oxidative stress-induced upregulation of ROS and the pro-apoptotic protein Bax. In contrast to the defective macro autophagy reported in the E50K OPTN over-expression cells, the transgenic animals showed increased mitophagosome formation. It is unclear whether the mitophagosome buildup is due to a defective mitophagic flux. However, the E50K OPTN expression in aged mice influenced the regulators of mitochondrial biogenesis with defects in mitochondrial fission/fusion cycles, causing fragmented mitochondria with higher cristae density and lower mitochondrial volume in RGC axons. These defects were replicated in RGC somas. Moreover, the mitochondrial transport and OXPHOS were not impaired in RGC axons. Therefore, the defects in mitochondrial biogenesis mediated by oxidative stress in E50K OPTN may also play a role in RGC death and the development of glaucoma (Shim et al., 2016).

In XFG, the accumulation of fibrillar ECM aggregates on the outflow pathway and proteostasis stress are more likely to induce mitochondrial dysfunction. Our lab has previously reported defective autophagy with reduced MMPT in the XFG patient-derived TFs (Want et al., 2016). Recently, we reported that the XFG TFs demonstrated mitochondrial dysfunction, characterized by impaired mitochondrial bioenergetics with decreased ATP, increased ROS, defective mitophagy flux, and altered mitochondrial ultrastructure with smaller mitochondria showing swollen cristae (Venkatesan et al., 2025). These defects are also observed in patients with other forms of glaucoma and animal models, indicating a common mechanism, such as IOP and oxidative stress involvement in glaucoma pathogenesis, as previously discussed. Interestingly, promoting mitophagy and enhancing mitochondrial biogenesis can reverse these mitochondrial defects in XFG TFs (Venkatesan et al., 2025). Future research will clarify the mechanism behind these mitochondrial defects in XFG disease.

### ER-mitochondria crosstalk in glaucoma

Mitochondrial-associated membranes (MAMs) are structures derived from the ER that act as contact points between the ER and mitochondria, facilitating the transport of lipids and Ca^2+^ to mitochondria. These structures act as friend and foe and are often regulated by the ER-UPR response, particularly by PERK. They are also implicated in autophagy by regulating autophagosome formation and mitochondrial fission-fusion events through MAMs, suggesting that ER stress directly affects mitochondrial function (Senft and Ronai, 2015; Kasi et al., 2025). In glaucoma, protein misfolding causes ER stress, and UPR-PERK activation may be the primary cause of oxidative stress, mitochondrial dysfunction, and the apoptosis of TM or RGC. While research has not established direct mechanistic links between ER stress and mitochondrial dysfunction in glaucoma, emerging evidence suggests that elevated Ca^2+^ uptake by mitochondria due to ER stress leads to RGC apoptosis (Hurley et al., 2022). This hypothesis is further supported by the observed recovery of RGCs when UPR activations are inhibited in animal models of glaucoma (Kasetti et al., 2020). Additionally, TM cells from patients with POAG show impaired Ca^2+^ regulation, which leads to the opening of mitochondrial permeability transition pores (He et al., 2008a). Thus, future studies in glaucoma are required to evaluate the role of MAMs in RGC degeneration.

### Biomarkers in glaucoma

The most effective treatment for glaucoma today is to reduce IOP (Wagner et al., 2022). However, glaucoma is often diagnosed late due to its slow progression, lack of warning signs, and asymptomatic early stages. Although IOP measurements and fundus photography are commonly used to identify glaucomatous damage, at least yearly visits to the Ophthalmologist are required to diagnose the condition before significant damage occurs (Schuster et al., 2020). This gap may be addressed using insights from GWAS studies, as genetic testing identified high-risk glaucoma variants (Ayala et al., 2023) in asymptomatic carriers who were at least 7 years younger than those diagnosed with clinical symptoms of glaucoma (Souzeau et al., 2017). Moreover, insights obtained from polygenic prediction (Craig et al., 2020) and genotype-phenotype studies offer prognostic value by enabling early diagnosis in individuals carrying certain high-risk genetic mutations associated with worse disease results.

Biomarkers in the AH that are associated with glaucoma have been heavily studied, including the identification of POAG-specific exosome characteristics. Glaucoma patients exhibited higher exosome particle counts and smaller sizes compared to controls, whereas higher exosome density was correlated with more severe visual field loss (Shin et al., 2024). In plasma, analysis of extracellular vesicle mRNAs in NTG patients revealed mitochondrial dysfunction and enrichment of central nervous system degenerative pathways relative to controls (Xu et al., 2024). Other cellular activities that correlate with disease progression include lipid peroxidation and total antioxidant activity (Hernandez-Martinez et al., 2016). Correspondingly, a recent review highlighted metabolic dysfunction in POAG. Key oxidative stress biomarkers were the enrichment of the arginine and proline metabolism pathway in both AH and plasma and the phenylalanine metabolism pathway in plasma (Golpour et al., 2025). Another metabolic study demonstrated that the concentration of small metabolites in the AH of glaucoma patients is altered. The imbalance affects membrane components, especially those of the mitochondria, suggesting that mitochondrial abnormalities either initiate or are a consequence of glaucoma (Lillo et al., 2022; Dammak et al., 2021). In addition to metabolic studies there are numerous other studies using AH and plasma from glaucoma patients. In XFG patients, the protein folding chaperone-clusterin was found to be significantly elevated in the AH compared to controls (Can Demirdogen et al., 2020). Another study demonstrated that IL-8 and Endothelin-1 are early biomarkers in the AH that are associated with XFS, prior to clinical manifestation of the disease, whereas Serum Amyloid A is associated with the later stage disease (Kadoh et al., 2025). Interestingly, another study supports the role of Endothelin-1 in glaucoma as AH from POAG and NTG patients relative to controls. These data suggest that dysregulation of vascular perfusion may have a role in the pathophysiology of POAG (Hedberg-Buenz et al., 2025). In a separate study that employed ELISA to measure inflammatory cytokines in the AH and compared POAG, XFG, and control groups, the researchers identified significant differences in several IL cytokines and growth factors across all groups. The key conclusion was that inflammation is closely linked to glaucoma, and specific cytokines may be associated with the severity of the condition (Gallardo Martin et al., 2025). Other studies have utilized glaucomatous primary cells for cell biological studies, leading to better characterization of the markers that define the glaucomatous outflow pathway (Yang et al., 2025). In-depth proteomic studies in cells have been used to understand the complex role of oxidative stress in age-related ocular disease. These data sets were compared to the AH from cataract and glaucoma patients, demonstrating RAD23B protein, involved in nuclear excision, repair, and proteasome regulation, as a potentially important glaucoma diagnostic marker (Rejas-Gonzalez et al., 2024). Overall, given the accessibility of AH and plasma for study, it is likely that diagnostic tools will become available to identify glaucoma patients prior to the onset of visual disability.

### Therapeutics targeting protein misfolding

Different therapeutic strategies have been suggested to enhance protein folding, inhibit aberrant interaction, and alleviate the glaucoma phenotype. Mutations in *MYOC* lead to POAG, resulting in a toxic gain of function caused by MYOC misfolding and aggregation, leading to ER stress-induced TM cell death. In addition to surgical approaches, therapeutics improve the folding and clearance of mutant MYOC, alleviate ER stress, and restore proteostasis to improve TM function and lower IOP. On that note, over-expression of the HSP chaperone GRP94 facilitates the triaging of mutant I477N MYOC, thereby preventing its degradation (Suntharalingam et al., 2012). Treatment with the GRP94 inhibitor 4-Br-BnIm enhances MYOC solubility and decreases TM cell toxicity (Stothert et al., 2014). A similar approach using small molecule chaperones, such as sodium-4-Phenylbutyrate (4-PBA), enhances the folding of MYOC variants by reducing interaction with chaperones, leading to greater secretion, fewer insoluble aggregates, less ER stress, and reduced PERK-ATF4-CHOP activation (Kasetti et al., 2016; Yam et al., 2007b; Zode et al., 2011). The 4-PBA can also reduce abnormal ECM deposition in a glucocorticoid-induced OHT model (Maddineni et al., 2021). Moreover, in the Y437H MYOC mouse model, treatment of 4-PBA for 5 weeks prevented nocturnal IOP elevation, RGCs loss, and ON degeneration (Zode et al., 2011). Similarly, Valdecoxib or Bexarotene treatments also reduced RGC apoptosis by preventing ER stress in different glaucoma models (Gao et al., 2022; Dheer et al., 2019). Furthermore, an *in vitro* study demonstrates that the efficacy of epicatechin gallate in preventing the aggregation of the OLF domain of MYOC into amyloid fibrils shows promise for future drug discovery studies (Sharma et al., 2022). A novel antibody-based therapy that showed promise in clearing misfolded proteins associated with protein-folding diseases is being investigated for *MYOC* pathogenic variants. Anti-OLF antibodies effectively recognize and degrade intracellular insoluble aggregated mutant MYOC through autophagy in *in vitro* cell line models, indicating potential for future gene therapy (Ma et al., 2025). This aligns with the OHT model, which demonstrated a decrease in IOP in animal models after inducing autophagic flux to break down mutant MYOC (Kasetti et al., 2021). The CRISPR-Cas9-based genome editing of the *MYOC* OHT mouse model also showed promising therapeutic results, as it reduced ER stress and led to lower IOP in the animals, improving TM tissue homeostasis and preventing further glaucomatous damage (Jain et al., 2017; Patil et al., 2024). Additionally, restoring TM cell function through stem cell-based transplantation approaches offers promising alternative therapies for glaucoma (Xiong et al., 2021; Zhu et al., 2016).

In OPTN-based NTG, drugs targeting TBK1 were effective in enhancing the solubility and neuroprotection of E50K OPTN. BX795, an aminopyrimidine chemical inhibitor, is known to inhibit the kinase activity of TBK1. Amlexanox is an anti-inflammatory, antiallergic immunomodulator that inhibits IKKε and TBK1 by competing for ATP binding to the enzyme. Since the TBK1 interaction is enhanced in E50K OPTN, treatment of both BX795 and Amlexanox in E50K OPTN cells increased the mutant solubility by limiting its interaction with TBK1. Additionally, a high dose of Amlexanox treatment in an E50K OPTN knock-in mouse model suppressed RGC layer thinning and improved neuroprotection without any adverse reactions in the animals (Minegishi et al., 2013; Minegishi et al., 2016). Although the TBK1 interaction is reduced in the case of M98K OPTN, the BX795 treatment rescued RGC-5 cell death by inhibiting S177 phosphorylation (Sirohi et al., 2015). Thus, the interaction of TBK1 with OPTN variants could serve as a potential treatment for OPTN-based NTG (Table 1). While strategies to properly fold pathogenic protein variants present promising options for treating POAG and NTG, such therapeutics have not been explored in XFG disease. Nevertheless, existing insights in XFG indicate that therapies designed to boost autophagy for better cellular proteostasis could represent a valuable approach. Nonetheless, a peptide-based method for the *ex vivo* extraction of fibrillar aggregates from human lens capsules demonstrates potential as a therapeutic approach (Ghaffari Sharaf et al., 2022).

### Therapeutics improving mitochondrial function

Due to the high energy demands of retinal tissue, mitochondrial dysfunction in glaucoma leads to RGC death. Several blood and AH-based non-invasive and invasive biomarker approaches, which target DNA analysis for mtDNA mutations, copy number variations (Vallabh et al., 2024), and mitochondrial oxidative/redox stress biomarkers, show promising diagnostic and prognostic value for glaucoma patients (Sorkhabi et al., 2011; Hondur et al., 2017; Takayanagi et al., 2020). Subsequent research has indicated that racial differences in oxidative stress biomarkers and distinct pathological characteristics offer prognostic insights for the targeted populations (Wu et al., 2022). Thus, these approaches could help identify at-risk patients early, before disease progression.

While mechanistic insights into mitochondrial dysfunction in glaucomatous neurodegeneration are still being developed, various therapeutic approaches to enhancing mitochondrial function have been proposed for glaucoma. These interventions focus on improving RGCs and ONH health by decreasing oxidative stress and enhancing mitochondrial function in glaucoma models. Antioxidant supplementation is an emerging therapy to reduce oxidative stress and protect RGCs. Coenzyme Q10 is an antioxidant cofactor, and its administration in the rat IOP model promoted RGC survival by blocking the apoptotic pathway (Ju et al., 2018). As we age, NAD^+^ levels decrease. However, using NAD^+^ precursors like nicotinamide and nicotinamide ribose is gaining attention as promising complementary therapies, including glaucoma. In glaucomatous animal models, treatment with NAD^+^ precursors demonstrated a protective effect on preserving RGCs and ONH by preventing mitochondrial damage (Williams et al., 2017; Zhang et al., 2021b). Strategies focusing on improving mitophagy to clear the damaged mitochondria are also explored in glaucoma. In a rat glaucoma model, the over-expression of the mitophagy modulator Parkin using adeno-associated virus 2 (AAV2) resulted in a notable reduction in RGC death, accompanied by a minor decrease in IOP (Dai et al., 2018). A similar approach, using AAV2-OPA1 overexpression in a rat glaucoma model, resulted in healthier mitochondria by promoting Parkin expression and protecting against RGC loss (Hu et al., 2018). Similarly, in E50K OPTN human stem cell-derived RGCs, inhibiting TBK1 with BX795 boosts mitochondrial biogenesis, enhances ATP production, and addresses various mitochondrial problems (Table 1) (Surma et al., 2023). In our lab, we recently demonstrated that treating XFG TFs with the mitophagy inducer Urolithin A and nicotinamide ribose enhanced mitochondrial bioenergetics and decreased ROS production (Venkatesan et al., 2025). Thus, improving mitochondrial health, which is often compromised by aging, genetic factors, and disease pathophysiology, may help slow the progression of glaucoma.

## Discussion

Glaucoma is a multifaceted disease influenced by genetic, environmental, and lifestyle factors. While its severity varies across subtypes, emerging evidence indicates that protein misfolding is a common underlying factor in POAG, NTG, and XFG. Advances in identifying risk alleles and experimental studies focused on molecular mechanisms suggest that the misfolding of mutant proteins causes toxicity by triggering increased ER stress, activating the UPR, and disrupting protein degradation due to altered cellular localization and function. The expression of these variant proteins impacts mitochondrial function, leading to elevated ROS levels, altered mitochondrial morphology, reduced ATP production, and impaired mitophagy, which ultimately contribute to RGC degeneration. Therapeutic strategies using chemical chaperones to enhance protein folding and antioxidant supplementation to boost mitochondrial function demonstrate promising results in glaucoma models. These crucial findings arise from primary patient samples, glaucomatous animal models, and *in vitro* overexpression of genetic variants. Further exploring gene-gene and gene-environment interactions remains essential, extending beyond epidemiological studies to enhance personalized medicine and disease management. Despite the development of novel therapies in the pre-clinical stage, there remains a pressing need for further research into the molecular mechanisms linking ER stress and mitochondrial dysfunction to the onset and progression of glaucoma.

## Glossary

4-PBA: Sodium-4-Phenylbutyrate
AAV2: Adeno-associated virus 2
AH: Aqueous humor
ALS: Amyotrophic lateral sclerosis
CC: Coiled-coil domain
CNX: Calnexin
CRT: Calreticulin
D2: DBA/2J
ECM: Extracellular matrix
ER: Endoplasmic reticulum
ERAD: ER-associated degradation
ERCa^2+^: Endoplasmic reticulum calcium
ERQC: ER protein quality control
ETC: Electron transport chain
GWAS: Genome-wide association studies
IDR: Intrinsically disordered region
IMM: Inner mitochondrial membrane
IOP: Intraocular pressure
JOAG: Juvenile open-angle glaucoma
LOXL1: Lysyl oxidase-like one
LZ: Leucine Zipper
MAM: Mitochondrial-associated membrane
MCU: Mitochondrial calcium uniporter
MitCa^2+^: Mitochondrial calcium
MMPT: Mitochondrial membrane potential
mtDNA: Mitochondrial DNA
MYOC: Myocilin
NGS: Next-generation sequencing
NTG: Normal tension glaucoma
OHT: Ocular hypertension
OLF: Olfactomedin
OMM: Outer mitochondrial membrane
ON: Optic nerve
ONH: Optic nerve head
OPTN: Optineurin
OXPHOS: Oxidative phosphorylation
POAG: Primary open-angle glaucoma
PTM: Post-translational modification
PTP: Permeability transition pore
RGCs: Retinal ganglion cells
ROS: Reactive oxygen species
SC: Schlemm’s canal
TF: Tenon fibroblast
TM: Trabecular meshwork
UBD: Ubiquitin-binding domain
UPR: Unfolded protein response
WT: Wild-type
XFG: Exfoliation glaucoma
